# How Does Combined Resection Affect the Clinical Outcomes After Laparoscopic Surgery for Serosa-Positive Gastric Cancer?: A Retrospective Cohort Study to Investigate the Short-Term Outcomes of Laparoscopic Combined Resection in Patients With T4b Gastric Cancer

**DOI:** 10.3389/fonc.2019.01564

**Published:** 2020-01-30

**Authors:** Chang Min Lee, San Lee, Danbi Lee, Sungsoo Park

**Affiliations:** ^1^Department of Surgery, Korea University College of Medicine, Seoul, South Korea; ^2^Department of Surgery, Korea University Medical Center Ansan Hospital, Ansan, South Korea; ^3^Department of Surgery, Korea University Medical Center Anam Hospital, Seoul, South Korea

**Keywords:** combined resection, gastric cancer, laparoscopic, T4a, T4b

## Abstract

**Background:** Only few surgeons have tried to perform laparoscopic combined resection for T4b gastric cancer. The purpose of this study was to investigate the feasibility of laparoscopic combined resection through a comparison of the clinical outcomes between cT4a and cT4b cases.

**Methods:** We reviewed the medical charts of patients who underwent laparoscopic gastrectomy for clinically T4 gastric cancer from May 2014 and July 2018. During this period, 62 patients with serosa-positive gastric cancer underwent laparoscopic curative surgery. The patients were divided into the following groups: patients who underwent gastrectomy and combined resection for the invaded organs (combined resection group) and those who did not undergo combined organ surgery (gastrectomy only group). Clinical outcomes were compared between the gastrectomy only and combined resection groups.

**Results:** Of 62 patients included in this study, 43 and 19 patients were included in the gastrectomy only and combined resection groups, respectively. The operation time was significantly longer in the combined resection group (364.6 ± 102.5 vs. 247.7 ± 66.1 min; *p* < 0.001). The incidence of grade ≥ III complications was comparable between the groups (26.3% vs. 11.6%; *p* = 0.147). The time from the first operation to the initiation of adjuvant chemotherapy showed no statistically significant difference between the groups (48.1 ± 45.4 days vs. 31.6 ± 9.2; *p* = 0.134).

**Conclusions:** Focusing on the high quality of image and new devices of laparoscopic surgery, it is necessary to re-evaluate the oncologic outcomes of combined resection for T4b gastric cancer.

## Introduction

R0 resection is the mainstay to achieve the survival benefit in patients with gastric adenocarcinoma. This principle should be also considered as a significant endpoint in the treatment for locally advanced cases; therefore, the current treatment guideline suggested combined resection in T4b gastric cancer. However, regarding this issue, several representative trials showed that combined organ resection resulted in a high rate of morbidities after curative surgery for advanced gastric cancer (AGC). Cuschieri et al. reported that combined pancreatectomy and splenectomy to achieve D2 resection increased the morbidity and mortality rates after gastric cancer surgery (1). Similarly, combined organ resection might be attributed to the higher morbidity rate of patients undergoing D2 dissection than D1 dissection according to the result of a Dutch trial (2). Given that postoperative morbidity is known to be correlated with oncologic outcomes, many concerns in performing combined surgery for patients with T4b disease have existed.

However, it is remarkable that all of these data have been acquired from the clinical experiences of open surgery for gastrectomy. To date, it has been difficult to apply the minimally invasive procedures in patients with AGC. Although several trials have been planned for investigating the oncologic safety of laparoscopic surgery in patients with AGC, many concerns still exist in the minimally invasive approach for serosa-positive disease. Thus, it has taken quite a long time to apply laparoscopic combined resection in patients with T4b cases.

These reluctances to the laparoscopic approach for AGC seem to be contradictory to the known advantages of minimally invasive approaches. For decades, many trials have proved that adjuvant chemotherapy (AC) showed a significant prognostic effect in patients undergoing curative gastrectomy (3, 4). With regard to this issue, it is necessary to consider the key characteristics of laparoscopic surgery. The fast recovery resulted from the reduced wound size, which enables the patients to undergo AC in a timely period. Actually, even though we achieve R0 resection in patients with AGC, the late induction of AC may cause a poor oncologic outcome (5).

In T4b diseases that necessitate combined organ resection, laparoscopic surgery is more effective than open surgery in terms of reducing the abdominal wound size. Although it can be necessary to extend the abdominal incision for combined resection during open surgery, laparoscopic combined resection requires only the addition of no or several port incisions.

For the recent years, we accumulated the clinical experience of laparoscopic surgery for serosa-positive gastric cancer. Of these, some patients with cT4b diseases underwent laparoscopic combined resection. Therefore, in this study, we investigated the feasibility of laparoscopic combined resection through a comparison of the clinical outcomes between cT4a and cT4b cases.

## Methods

### Study Design and Participants

**Figure 1:**
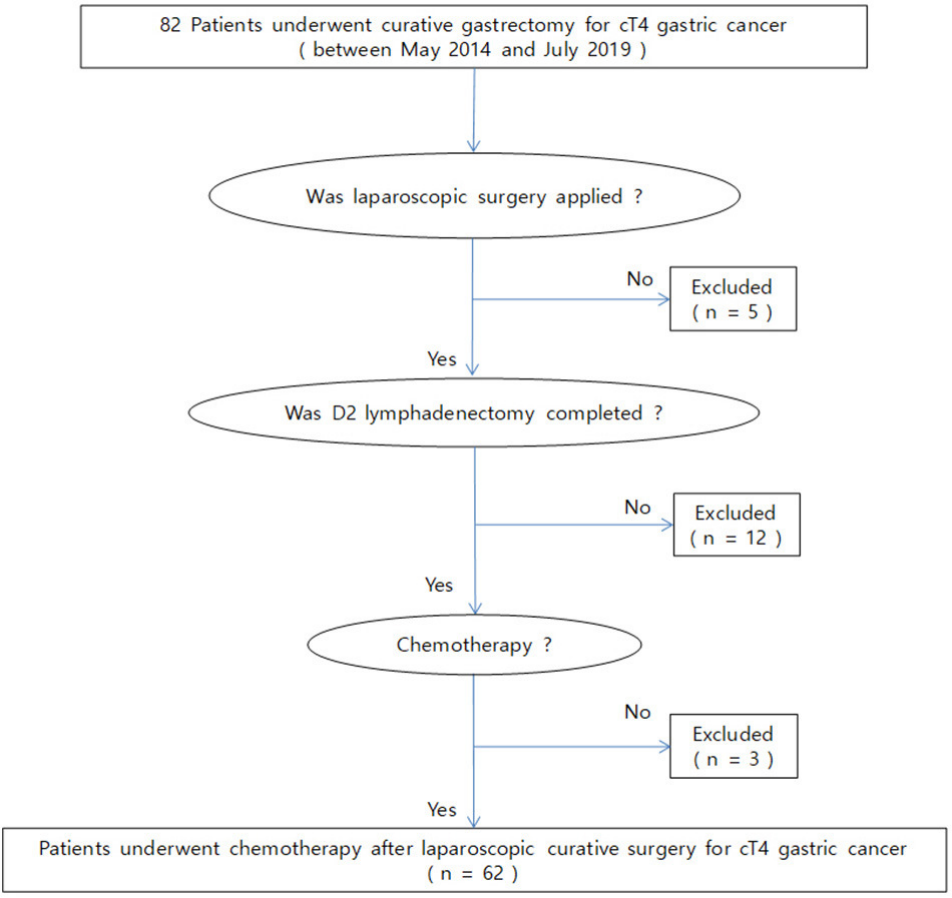
Schematic of this study.

This was a retrospective cohort study performed in a single institution. We reviewed the medical charts of patients who underwent laparoscopic gastrectomy for clinically T4 gastric cancer between May 2014 and July 2018. During this period, a total of 65 patients with serosa-positive gastric cancer underwent laparoscopic curative surgery. Of these, 62 patients were treated with AC (Figure 1). According to whether combined organ resection was performed, the patients were divided into the following two groups: patients who underwent gastrectomy and combined resection for the invaded organs (named the combined resection group) and those who did not undergo combined organ surgery (named the gastrectomy only group). Clinical outcomes were compared between the gastrectomy only and combined resection groups.

The approval to perform research on human subjects in this study was provided by the Institutional Review Board of Korea University Medical Center Ansan Hospital (registration number: 2019AS0205). This study adhered to the tenets of the Declaration of Helsinki.

### Procedures

All surgical procedures were performed by one surgeon (CML), who had been trained in a high-volume center performing more than 500 laparoscopic gastric cancer surgeries per year.

In the operating room, the procedure was performed under general anesthesia with the patient placed on the bed with both legs abducted. The bed was adjusted to create a reverse Trendelenburg position for the patient. The operator sat on the right side of the patient, while the scopist was positioned between the patient's legs.

A 12-mm channel trocar was inserted through a transumbilical incision using the Hasson's method (6). After a pneumoperitoneum was formed with carbon dioxide at a pressure of 15 mmHg, a flexible scope was inserted through this umbilical port. Under the guidance of flexible scope, one 5-mm channel trocar was established on the right subcostal margin and the other 12-mm channel trocar on the right midclavicular line. Additionally, two 5-mm channel trocars were established on the left subcostal margin and left midclavicular line.

The falciform ligament and the left lobe of the liver were raised toward the cephalad direction by combined suture retraction (7).

Lymphadenectomy for curative distal gastrectomy was accomplished based on the criteria of the Japanese Gastric Cancer Treatment Guidelines 2010 (ver. 3) (8). We performed D2 lymphadenectomy in all of the patients who were preoperatively diagnosed with clinically T4.

**Figure 2:**
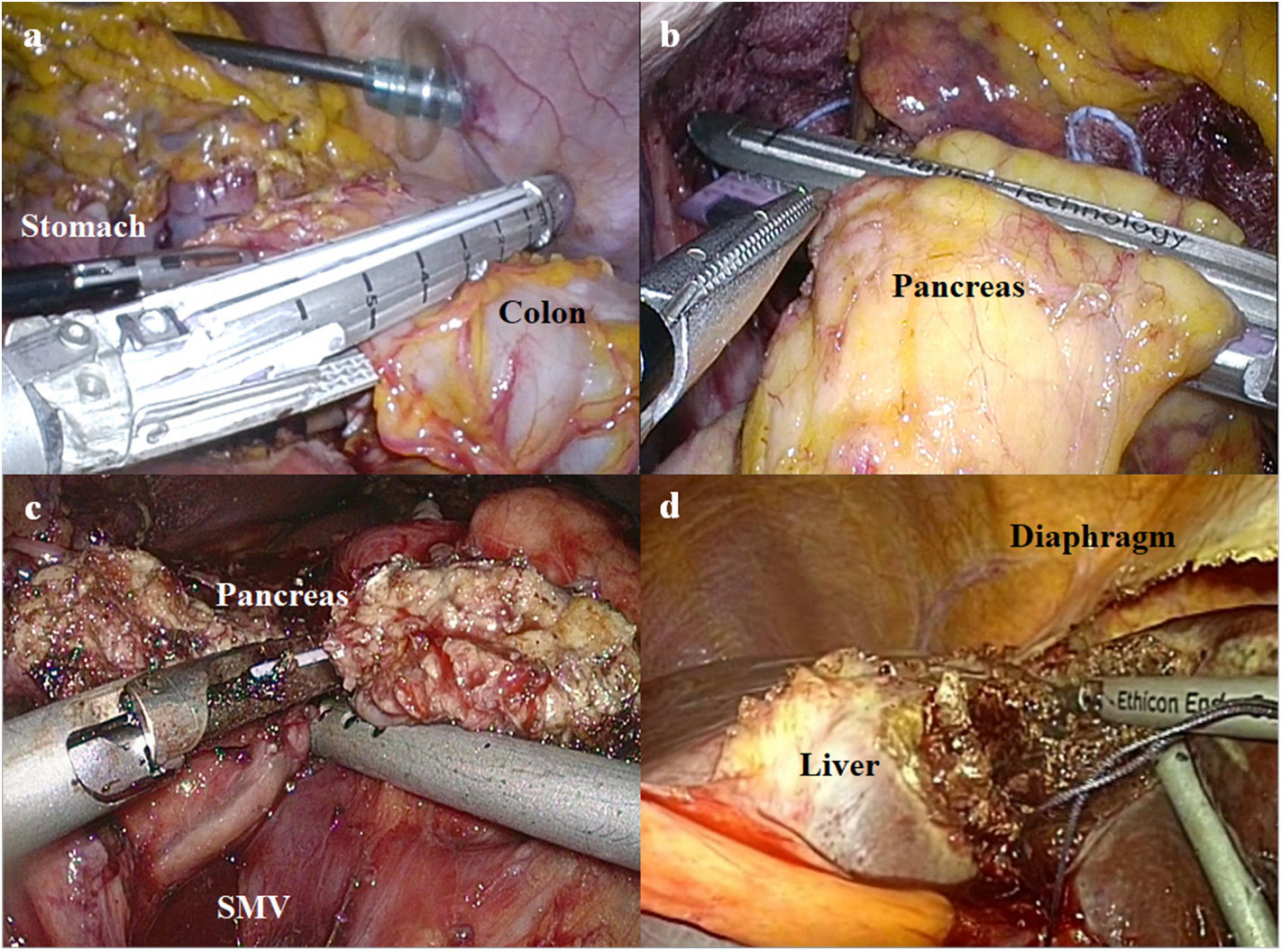
The procedures of combined resection for advanced gastric cancer invading the adjacent organs. **(a)** Laparoscopic linear stapler is used to resect the colon invaded by advanced gastric cancer. **(b)** Distal pancreatectomy is performed using a laparoscopic linear stapler. **(c)** Pancreas is transected using an ultrasonic energy device during pancreaticoduodenectomy (SMV, superior mesenteric vein). **(d)** Ultrasonic energy device is used to resect the liver invaded by advanced gastric cancer.

After the completion of lymphadenectomy, the stomach and adjacent organs (particularly in the combined resection group) were resected. The gastrointestinal or hepatopancreaticoenteric continuity was recovered according to the following types of the resected organs: (i) in cases of Billroth II anastomosis after subtotal gastrectomy, Braun anastomosis was also performed to reduce bile reflux to the remnant stomach. All of the gastrointestinal anastomoses were performed with laparoscopic linear staplers; (ii) in cases of Roux-en-Y esophagojejunostomy after total gastrectomy, jejunojejunostomy was made at the 45-cm distal point from the esophagojejunostomy. In all of these cases, the mesentery defect was closed using non-absorbable knotless barbed sutures. All of the gastrointestinal anastomoses were performed with laparoscopic linear staplers; (iii) in cases invading the transverse colon, the involved segment was resected using laparoscopic linear staplers (Figure 2a). Colo-colic anastomosis was performed by overlap stapling using laparoscopic linear staplers; (iv) in cases invading the body or tail of the pancreas, distal pancreatectomy was performed using laparoscopic linear staplers (Figure 2b). Reinforcement sutures were performed using knotless barbed sutures; (v) in cases invading the head of the pancreas, pancreaticoduodenogastric resection was performed (Figure 2c). To restore the bilio-pancreatico-intestinal continuity, pancreaticojejunostomy and hepaticojejunostomy were performed by hand-sewing. Billroth II anastomosis was performed for the recovery of gastrointestinal continuity; and (vi) in cases invading the liver, the resection ranges were determined according to the location and size of the involved segments. If the tumor extensively involved both segments 2 and 3, left lateral sectionectomy was performed (Figure 2d). If the invaded lesion was localized in segment 2 or 3, non-anatomical resection was performed.

### Assessments

Demographic data, including age, sex, body mass index (BMI), and American Society of Anesthesiologists (ASA) score, were obtained from all enrolled patients. In addition, clinical outcomes, including the operation time, conversion to open surgery, reconstruction method, resected organs, postoperative hospital stay, time to the first semi-blend diet, postoperative complications, and the time from the first operation to the initiation of AC were also investigated. Postoperative complications were classified based on the Clavien-Dindo classification of surgical complications (9).

We also investigated the pathologic results, including tumor depth, and number of retrieved and metastatic lymph nodes.

As a subgroup analysis, the patients in the combined resection group were subdivided into the following three groups: patients who underwent splenectomy (named as SP group), those who underwent distal pancreatectomy and splenectomy (named as DP group), and the others (named as OT group). Clinical outcomes were compared between the SP, DP, and OT groups.

### Analysis

Patients with and without undergoing combined resection were compared using chi-square test or Fisher's exact test for categorical data and Student's *t*-test or Mann–Whitney *U* test or one-way ANOVA for continuous data without normal distribution. In the two-tailed tests, a *p* ≤ 0.05 was considered statistically significant. Statistical analysis was performed using SPSS 24.0 (SPSS Inc., Chicago, IL, USA).

## Results

Of 62 patients included in this study, 43 patients had clinically T4a disease (gastrectomy only group) and 19 had clinically T4b (combined resection group). All of the patients in the combined resection group underwent laparoscopic en bloc resection of the stomach and adjacent organs.

### Demographic Data

The mean age of the patients was 62.6 ± 13.1 years, and the gastrectomy only group was significantly older (65.9 ± 13.1 vs. 55.2 ± 9.6 years; *p* = 0.001). The sex ratio was not different between two groups (*p* = 0.172). Baseline BMI was significantly higher in the gastrectomy only group (23.0 ± 3.1 vs. 21.0 ± 3.5 kg/m^2^; *p* = 0.031). Most of the patients' pathologic T stages were similar to the clinical T stage before the surgery (83.9%), with the exception of 10 patients (seven with combined resection and three with gastrectomy only).

### Clinical Outcomes

The clinical outcomes showed that the operation time was significantly longer in the combined resection group (247.7 ± 66.1 vs. 364.6 ± 102.5 min; *p* < 0.001). The time to the first semi-blend diet and the length of hospital stay were also significantly longer in the combined resection group (Table 1).

**Table 1:** Characteristics of patients who underwent laparoscopic gastrectomy with D2 lymphadenectomy for clinical T4 gastric cancer (*n* = 62).

	**Gastrectomy only group (*n* = 43)**	**Combined resection group (*n* = 19)**	***p***
Age	65.9 ± 13.1	55.2 ± 9.6	0.001
Sex (Male:Female)	2.1:1	5.3:1	0.172
BMI	23.0 ± 3.1	21.0 ± 3.5	0.031
ASA score (%)			0.710
1	4 (9.3)	3 (15.8)	
2	31 (72.1)	12 (63.2)	
3	8 (18.6)	4 (21.1)	
The type of surgery (DG:TG)			<0.001
DG	31 (72.1)	4 (21.1)	
TG	12 (27.9)	15 (78.9)	
The operation time (min)	247.7 ± 66.1	364.6 ± 102.5	<0.001
Suture for intraoperative bleeding (%)	5 (11.6)	6 (31.6)	0.135
Portal vein injury	3 (7.0)	3 (15.8)	
Splenic artery injury	0	2 (10.5)	
Gastroduodenal artery injury	1 (2.3)	1 (5.3)	
Proper hepatic artery injury	1 (2.3)	0	
Pathologic T stage (%)			<0.001
pT1	0	0	
pT2	3 (7.0)	0	
pT3	0	1 (5.3)	
pT4a	40 (93.0)	6 (31.6)	
pT4b	0	12 (63.2)	
Number of retrieved lymph nodes	43.1 ± 22.2	54.4 ± 27.3	0.090
Number of metastatic lymph nodes	10.3 ± 13.4	7.5 ± 7.8	0.400
Pathologic N stage			0.601
pN0	8 (18.6)	3 (15.8)	
pN1	6 (14.0)	5 (26.3)	
pN2	9 (20.9)	5 (26.3)	
pN3a	11 (25.6)	3 (15.8)	
pN3b	9 (20.9)	2 (10.5)	
The length of hospital stays (day)	15.2 ± 5.4	36.0 ± 40.9	0.040
Morbidity (%)	9 (20.9%),	12 (63.2%)	0.001
Severe morbidity (≥grade III) (%)	5 (11.6%)	5 (26.3%)	0.147
The details of morbidity (%)			0.014
Fluid collection	4 (9.3)	8 (42.1)	
Duodenal stump leakage	2 (4.7)	1 (5.3)	
Anastomosis leakage	0	1 (5.3)	
Pneumonia	2 (4.7)	1 (5.3)	
Bleeding	1 (2.3)	0	
Afferent loop syndrome	0	1 (5.3)	
The time to adjuvant chemotherapy	31.6 ± 9.2	48.1 ± 45.4	0.134

*BMI, body mass index; ASA score, score graded by the American Society of Anesthesiologists physical status classification; DG, distal gastrectomy; TG, total gastrectomy*.

The combined resection group experienced postoperative complications more frequently (63.2%) than the gastrectomy only group (20.9%); however, the incidence of grade ≥ III was comparable between the groups (11.6% in the gastrectomy only group vs. 26.3% in the combined resection group; *p* = 0.147). In addition, the time from the first operation to the initiation of AC showed no statistically significant difference between the groups (31.6 ± 9.2 in gastrectomy only group vs. 48.1 ± 45.4 days in combined resection; *p* = 0.134) (Table 1). There was no complication that resulted in mortality in both groups.

### Detailed Information for the Combined Resection Group

In the combined resection group, three transverse colon invasions, six pancreas invasions (one head, three body, and two tail), five liver invasions, seven spleen invasions, and one lung invasion were noted in the laparoscopic images (Table 2).

**Table 2:** Clinicopathologic data of the patients who underwent laparoscopic combined resection.

**Serial number**	**Age**	**Sex**	**BMI**	**ASA score**	**Tumor location**	Tumor size (cm)^*^	**Type of gastrectomy**	**Invaded organ in laparoscopic view**	**Procedures for invaded organs**	**Operation time (min)**	**EBL (ml)**	**Hospital stay (days)**	**Time to the first SBD (days)**	**Morbidity**	**C-D grade**
1	58	Male	23.0	1	Low body	7.0	TG	Transverse colon	Segmental resection of transverse colon	282	50	13	6	None	0
2	50	Male	18.2	2	Antrum	7.0	DG	Pancreas (head)	Pancreaticoduodenectomy	650	500	34	9	Fluid collection	IIIa
3	73	Male	15.3	1	High body	6.0	TG	Liver	LLS	463	100	15	7	None	0
4	59	Male	17.6	2	From high body to distal esophagus	10.0	TG	Liver, lung	Splenectomy, LLS, wedge resection of lung	487	100	49	42	Anastomotic leakage of esophagojejunostomy	IIIa
5	40	Male	17.0	2	Antrum	4.5	DG	Liver	Wedge resection of liver	282	100	39	9	Leakage of duodenal stump	II
6	55	Male	20.0	2	High body	9	TG	Spleen	Splenectomy	358	250	20	8	None	0
7	51	Male	22.6	2	High body	6.5	TG	Spleen	Splenectomy	350	350	15	8	Afferent loop syndrome	IIIa
8	53	Male	20.4	1	High body	8	TG	Spleen	Splenectomy	402	50	43	4	Fluid collection	II
9	55	Male	23.5	2	High body	6.5	TG	Spleen	Splenectomy	273	50	22	9	Fluid collection	II
10	46	Male	22.1	2	From high body to cardia	6	TG	Pancreas (body)	DP, Splenectomy	412	450	22	8	Fluid collection	II
11	62	Male	22.0	2	Low body	4.5	DG	Liver	Wedge resection of liver	266	100	17	5	None	0
12	66	Male	26.1	3	From mid to high body	9	TG	Spleen	Splenectomy	369	300	182	10	Pneumonia	IVa
13	53	Male	24.3	2	High body	5	TG	Pancreas (body)	DP, splenectomy, segmental resection of transverse colon	397	50	16	5	None	0
14	45	Female	16.8	2	From mid to high body	12.5	TG	Spleen	Splenectomy.	340	100	11	9	None	0
15	41	Female	19.9	2	High body	6	TG	Spleen	Splenectomy	285	350	22	8	Fluid collection	II
16	71	Male	29.7	3	From cardia to distal esophagus	8	TG	Liver	Wedge resection of liver	319	100	13	6	None	0
17	69	Male	19.7	3	From high body to cardia	10	TG	Pancreas (tail), transverse colon	DP, splenectomy, segmental resection of transverse colon	391	100	22	12	Fluid collection	IIIa
18	52	Male	20.9	3	From low body to cardia	13	TG	Pancreas (body)	DP, splenectomy	293	450	52	7	Fluid collection	II
19	49	Female	29.3	2	From high body to cardia	10	TG	Pancreas (tail), transverse colon	DP, splenectomy	408	200	27	8	Fluid collection	II

*BMI, body mass index; ASA score, score graded by the American Society of Anesthesiologists physical status classification; EBL, estimated blood loss; C-D grade, grade by the Clavien-Dindo classification of surgical complications; TG, total gastrectomy; DG, distal gastrectomy; LLS, left lateral sectionectomy of liver; DP, distal pancreatectomy*.

* *These values are expressed as the longest diameter of the tumor*.

The postoperative complications in cases 8, 9, 10, and 19 appeared as intra-abdominal fluid collections, which were treated with the intravenous antibiotics (grade II by the Clavien-Dindo classification of surgical complications). Cases 2 and 17 also manifested the intra-abdominal fluid collection, but required the percutaneous abscess drainage (grade IIIa). Case 4 showed the anastomotic leakage of esophagojejunostomy, which was conservatively treated after endoscopic stent insertion (grade IIIa). Case 5 manifested duodenal stump leakage, which was treated with intravenous antibiotics (grade II). Case 12 was diagnosed with intractable pneumonia, which was treated in intensive care unit for more than 5 months; therefore, started AC treatment was started at 182 days postoperatively.

### Subgroup Analysis for the Combined Resection Group

The subgroup analysis (performed in the combined resection group) showed a statistically significant difference between the SP, DP, and OT groups in terms of the pathologic T stage. However, the operation time, the pathologic N stage, the length of hospital stays, morbidity, severe morbidity, and the time from the first operation to the initiation of AC did not differ between the three groups (Table 3).

**Table 3:** Subgroup analysis for combined resection group.

	**Splenectomy (*n* = 7)**	**Splenectomy and DP (*n* = 3)**	**Others (*n* = 9)**	***p***
Age	52.3 ± 8.0	49.0 ± 3.0	59.4 ± 10.8	0.164
Sex (Male:Female)	2.5:1	2:1	9:0	0.198
BMI	21.3 ± 3.0	24.1 ± 4.5	19.6 ± 3.2	0.170
ASA score (%)				0.869
1	1 (14.3)	0	2 (22.2)	
2	5 (71.4)	2 (66.7)	5 (55.6)	
3	1 (14.3)	0	2 (22.2)	
The type of surgery (DG:TG)				0.060
DG	0	0	4 (44.4)	
TG	7 (100)	3 (100)	5 (55.6)	
The operation time (min)	339.6 ± 45.9	371.0 ± 67.6	381.9 ± 141.6	0.733
Suture for intraoperative bleeding (%)				0.481
Portal vein injury	1 (14.3)	1 (33.3)	1 (11.1)	
Splenic artery injury	2 (28.6)	0	0	
Gastroduodenal artery injury	0	0	1 (11.1)	
Proper hepatic artery injury	0	0	0	
Pathologic T stage (%)				0.020
pT1	0	0	0	
pT2	0	0	0	
pT3	1 (14.3)	0	0	
pT4a	5 (71.4)	0	1 (11.1)	
pT4b	1 (14.3)	3 (100)	8 (88.9)	
Number of retrieved lymph nodes	65.3 ± 33.5	49.7 ± 13.3	47.4 ± 24.7	0.434
Number of metastatic lymph nodes	6.7 ± 9.8	8.3 ± 6.4	7.8 ± 7.4	0.949
Pathologic N stage				0.690
pN0	1 (14.3)	1 (33.3)	1 (11.1)	
pN1	3 (42.9)	1 (33.3)	1 (11.1)	
pN2	1 (14.3)	0	4 (44.4)	
pN3a	1 (14.3)	1 (33.3)	1 (11.1)	
pN3b	1 (14.3)	0	1 (11.1)	
The length of hospital stays (days)	45.0 ± 61.3	33.7 ± 16.1	29.8 ± 27.6	0.777
Morbidity (%)	5 (71.4)	3 (100)	4 (44.4)	0.191
Severe morbidity (≥grade III) (%)	3 (42.9)	0	2 (22.2)	0.344
The details of morbidity (%)				0.359
Fluid collection	3 (42.9)	3 (100)	2 (22.2)	
Duodenal stump leakage	0	0	1 (11.1)	
Anastomosis leakage	0	0	1 (11.1)	
Pneumonia	1 (14.3)	0	0	
Bleeding	0	0	0	
Afferent loop syndrome	1 (14.3)	0	0	
The time to adjuvant chemotherapy	59.7 ± 70.8	35.7 ± 3.8	43.1 ± 26.0	0.698

*DP, distal pancreatectomy; BMI, body mass index; ASA score, score graded by the American Society of Anesthesiologists physical status classification; DG, distal gastrectomy; TG, total gastrectomy*.

## Discussion

Laparoscopic surgery has been established as an effective modality for the surgical treatment of gastric cancer. Although it is still necessary to acquire the clinical evidences regarding laparoscopic combined surgery in patients with gastric cancer, the laparoscopic approach also has some advantages in terms of multi-organ resection. Most importantly, surgical trauma is minimized, because the diversity of the laparoscopic procedures can be expanded by adding several (sometimes no) port wounds. This characteristic has been known to be correlated with the fast recovery; therefore, the promising outcomes are expected in laparoscopic combined resection. Although many surgeons are concerned about whether all the procedures could not be realized with the laparoscopic approach, the recent advances in laparoscopic instruments (i.e., energy devices, surgical staplers, and high-resolution laparoscopes) have facilitated us to overcome the technical difficulty of laparoscopic procedures.

Nevertheless, only few surgeons have tried to perform laparoscopic combined resection for T4b gastric cancer (10). This tendency involves the following reasons. First, when gastric cancer shows the feature of T4b disease, the adjacent organs or tissues might be deprived of their inherent structures. Such deformities make it difficult to perform a laparoscopic approach; therefore, open surgery is generally preferred to control the risk from the unusual anatomy of T4b disease (i.e., the distorted flow of the named vessels, unexpected hemorrhage due to the neovascularization around the tumor, and ambiguous boundaries between the organs).

Meanwhile, another reason is associated with the current training systems for the surgeons. Nowadays, to acquire the qualified procedures for the oncologic principles, the surgeon's training program has been subdivided in Korea. Thus, most surgeons who had been trained for gastric cancer surgery might be unfamiliar with laparoscopic resection of the colon, pancreas, and liver. To solve these problems, some surgeons adopt the multidisciplinary approach for combined resection. However, unlike concomitant resection for the double primary malignancies, combined resection for T4b gastric cancer should be very organized that the main procedures cannot be distributed to each surgeon of the multidisciplinary team. Therefore, the foregut surgeons who usually deal with AGC should take the responsibility for en bloc resection of the invaded organs, which has been usually performed in open surgery.

Regarding this issue, we have prepared the clinical practice for a foregut surgeon to perform laparoscopic en bloc resection in patients with AGC invading the adjacent organs. Before the actual practice, we had organized many times multidisciplinary conference between the surgeons with different subspecialties in our institute. Nowadays, the well-developed video recording systems could provide high-quality images showing the details of laparoscopic surgery; therefore, our multidisciplinary conference has been operated based on the discussions regarding such video records. Moreover, in Korea, there had been some annual international conferences held by the Korean Society of Endoscopic and Laparoscopic Surgeons, in which many surgeons share the expertise from each subspecial division of laparoscopic surgery. From all of these programs outside and inside our institute, we have accumulated expertise in performing surgeries of the adjacent organs surrounding the stomach.

Nevertheless, there are several technically demanding points when performing combined surgery for T4b gastric cancer. First, such an advanced tumor makes the significant desmoplastic reaction around the major vessels; therefore, it can cause unexpected bleeding during the operation. In our results, there were many hemorrhagic events, all of which were controlled by laparoscopic suture. Even though the unexpected hemorrhage can happen during laparoscopic surgery for AGC, it can be even more dangerous to control the bleeding from the unaccustomed structures beyond the perigastric vascular anatomies. However, the recent technologic advances have enabled us to overcome the difficulties of laparoscopic surgery for AGC. Most of all, the high-resolution view of the current laparoscopic image induces us to comprehend the distorted anatomies around the tumor (11). Such a high-quality imaging system is expected to enhance the surgeons' eyes during gastric cancer surgery; therefore, it is possible that we may reach the stage that our ancestor surgeons could not achieve. Moreover, the diverse types of the energy devices allow us to perform the meticulous dissection over the desmoplastic adhesion.

**Figure 3:**
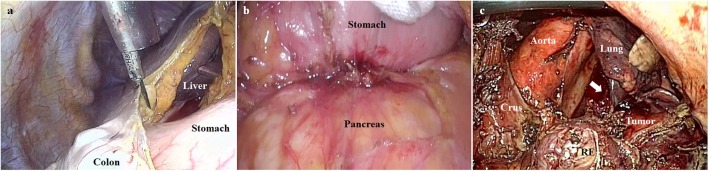
The poor surgical views due to the heavy tumor burden. **(a)** The surgical view can be limited by the heavy stomach (due to tumor weight or impacted food contents). **(b)** The pancreatic invasion can limit the exposure of the surgical field. **(c)** The surgical view can be limited by lung invasion (indicated by the white arrow) in case of esophagogastric junction cancer (RE, resected esophagus).

The next demanding point lies in the poor surgical view due to the heavy tumor burden. Since lymph node dissection should precede the removal of tumor-involved organs, the huge and heavier tumor causes significant obstacles to the surgical view. For instance, when we elevate the stomach to expose the lymphadenectomy field, the heavy stomach (due to tumor weight or impacted food contents) can restrict the surgical view (Figure 3a). Sometimes, tumor invasion also limits the exposure of the surgical field (Figure 3b). Additionally, in case of esophagogastric junction cancer, the surgical view can be limited by the chest organ invasion that is rarely encountered during gastric cancer surgery (Figure 3c). In our practice, the only strategy we applied in such conditions was to find the appropriate arrangement of the counter-traction. To ameliorate the clinical outcomes, it was necessary to establish an innovative method of overcoming tumor burden during lymphadenectomy.

Finally, combined resection itself can increase the postoperative morbidity rate regardless if the approach is laparoscopic or open. This issue should be solved to take the legitimacy of laparoscopic combined surgery, since the postoperative complications can delay the initiation of AC. In other words, it is necessary to show that the poor prognosis is not caused by the “laparoscopic combined resection” itself. In this study, no statistically significant difference was found between gastrectomy only and combined resection groups in terms of the time to the initiation of AC. Even though we investigated the small number of cases, it is expected that combined resection itself was not the only factor delaying the initiation of AC, because duodenal stump leakage, postoperative ileus, and complicated fluid collection can happen after the standard surgery for gastric cancer, even in early disease. Moreover, these parameters should be carefully interpreted in our results, because we have contrived ways to proceed with AC despite the postoperative complications, which are as follows: (1) we have intraoperatively inserted percutaneous transhepatic biliary drainage (PTBD) to prepare for postoperative pancreatic fistula (POPF) in patients undergoing pancreaticoduodenectomy (12); therefore, a patient in the combined resection group (case 2, Table 2) started treatment with AC at 52 days postoperatively with PTBD kept; (2) although a patient in the combined resection group (case 7, Table 2) had afferent loop syndrome, AC was started at 21 days postoperatively with PTBD kept. He recovered later without any re-operation; (3) a sump drain was routinely inserted to prepare for POPF in patients undergoing distal pancreatectomy; therefore, a patient in the combined resection group (case 18, Table 2) started AC treatment at 33 days postoperatively. All of these strategies have been adopted to avoid the delay of AC.

In conclusion, it is necessary to reboot the survival outcomes regarding gastric cancer surgery. Such trials can be supported by the results of CLASS-01 studies, in which non-inferiority of laparoscopic surgery for AGC was shown (13). Although laparoscopic surgery for AGC should be verified in the various aspects, it obviously provides the new surgical view and skills for gastric cancer surgery. These items may provide us with a chance to re-evaluate the oncologic outcomes of combined resection for T4b gastric cancer. In addition, if we consider the ways to abolish the correlation between postoperative complication and chemotherapy, all of these strategies should be based on the surgeon performing AC (14).

## Data Availability Statement

The raw data supporting the conclusions of this article will be made available by the authors, without undue reservation, to any qualified researcher.

## Ethics Statement

The studies involving human participants were reviewed and approved by the Institutional Review Board of Korea University Medical Center Ansan Hospital. The patients/participants provided their written informed consent to participate in this study.

## Author Contributions

CL designed the main concept of this study, and write the manuscript. SL collected the data. DL interpreted the data. SP verified all the contents of the study and manuscript.

### Conflict of Interest

The authors declare that the research was conducted in the absence of any commercial or financial relationships that could be construed as a potential conflict of interest.

## References

[1] Cuschieri A Fayers P Fielding J Craven J Bancewicz J Joypaul V. Postoperative morbidity and mortality after D1 and D2 resections for gastric cancer: preliminary results of the MRC randomised controlled surgical trial. The Surgical Cooperative Group. Lancet. (1996) 347:995–9. 10.1016/S0140-6736(96)90144-0 8606613

[2] Bonenkamp JJ Songun I Hermans J Sasako M Welvaart K Plukker JT. Randomised comparison of morbidity after D1 and D2 dissection for gastric cancer in 996 Dutch patients. Lancet. (1995) 345:745–8. 10.1016/S0140-6736(95)90637-1 7891484

[3] Paoletti X Oba K Burzykowski T Michiels S Ohashi Y Pignon JP. Benefit of adjuvant chemotherapy for resectable gastric cancer: a meta-analysis. JAMA. (2010) 303:1729–37. 10.1001/jama.2010.534 20442389

[4] Sakuramoto S Sasako M Yamaguchi T Kinoshita T Fujii M Nashimoto A. Adjuvant chemotherapy for gastric cancer with S-1, an oral fluoropyrimidine. N Engl J Med. (2007) 357:1810–20. 10.1056/NEJMoa072252 17978289

[5] Park HS Jung M Kim HS Kim HI An JY Cheong JH. Proper timing of adjuvant chemotherapy affects survival in patients with stage 2 and 3 gastric cancer. Ann Surg Oncol. (2015) 22:224–31. 10.1245/s10434-014-3949-2 25081339

[6] Hasson HM. A modified instrument and method for laparoscopy. Am J Obstet Gynecol. (1971) 110:886–7. 10.1016/0002-9378(71)90593-X 4254516

[7] Shabbir A Lee JH Lee MS Park DJ Kim HH. Combined suture retraction of the falciform ligament and the left lobe of the liver during laparoscopic total gastrectomy. Surg Endosc. (2010) 24:3237–40. 10.1007/s00464-010-1118-7 20526627

[8] Japanese Gastric Cancer Association Japanese gastric cancer treatment guidelines 2010 (ver. 3). Gastric Cancer. (2011) 14:113–23. 10.1007/s10120-011-0042-4 21573742

[9] Clavien PA Barkun J de Oliveira ML Vauthey JN Dindo D Schulick RD. The Clavien-Dindo classification of surgical complications: five-year experience. Ann Surg. (2009) 250:187–96. 10.1097/SLA.0b013e3181b13ca2 19638912

[10] Lee CM Rao J Son SY Ahn SH Lee JH Park DJ. Laparoscopic gastrectomy for gastric cancer with simultaneous organ resection. J Laparoendosc Adv Surg Tech A. (2013) 23:861–5. 10.1089/lap.2013.0081 23968253

[11] Shinohara T Uyama I Kanaya S Inaba K Isogaki J Horiguchi A. Totally laparoscopic pancreaticoduodenectomy for locally advanced gastric cancer. Langenbecks Arch Surg. (2009) 394:733–7. 10.1007/s00423-009-0492-x 19404673

[12] Lee CM Suh YJ Yoon SY. Retrograde installation of percutaneous transhepatic negative-pressure biliary drainage stabilizes pancreaticojejunostomy after pancreaticoduodenectomy: a retrospective cohort study. World J Surg Oncol. (2019) 17:101. 10.1186/s12957-019-1645-1 31196100 PMC6567420

[13] Yu J Huang C Sun Y Su X Cao H Hu J. Effect of laparoscopic vs open distal gastrectomy on 3-year disease-free survival in patients with locally advanced gastric cancer: the CLASS-01 randomized clinical trial. JAMA. (2019) 321:1983–92. 10.1001/jama.2019.5359 31135850 PMC6547120

[14] Min JS Lee CM Choi SI Seo KW Park DJ Baik YH. Who can perform adjuvant chemotherapy treatment for gastric cancer? a multicenter retrospective overview of the current status in Korea. J Gastric Cancer. (2018) 18:264–73. 10.5230/jgc.2018.18.e29 30276003 PMC6160523

